# HIV integrase variability and genetic barrier in antiretroviral naïve and experienced patients

**DOI:** 10.1186/1743-422X-8-149

**Published:** 2011-03-31

**Authors:** Antonio Piralla, Stefania Paolucci, Roberto Gulminetti, Giuditta Comolli, Fausto Baldanti

**Affiliations:** 1 Molecular Virology Unit, Virology and Microbiology Department, Fondazione IRCCS Policlinico San Matteo, 27100 Pavia, Italy; 2 Institute of Infectious Diseases, Fondazione IRCCS Policlinico San Matteo, 27100 Pavia, Italy; 3 Experimental Research Laboratories, Biotechnology Area, Fondazione IRCCS Policlinico San Matteo, 27100 Pavia, Italy

## Abstract

**Background:**

HIV-1 integrase (IN) variability in treatment naïve patients with different HIV-1 subtypes is a major issue. In fact, the effect of previous exposure to antiretrovirals other than IN inhibitors (INI) on IN variability has not been satisfactorily defined. In addition, the genetic barrier for specific INI resistance mutations remains to be calculated.

**Methods:**

IN variability was analyzed and compared with reverse transcriptase (RT) and protease (PR) variability in 41 treatment naïve and 54 RT inhibitor (RTI) and protease inhibitor (PRI) experienced patients from subjects infected with subtype B and non-B strains. In addition, four HIV-2 strains were analyzed in parallel. Frequency and distribution of IN mutations were compared between HAART-naïve and RTI/PI-experienced patients; the genetic barrier for 27 amino acid positions related to INI susceptibility was calculated as well.

**Results:**

Primary mutations associated with resistance to INI were not detected in patients not previously treated with this class of drug. However, some secondary mutations which have been shown to contribute to INI resistance were found. Only limited differences in codon usage distribution between patient groups were found. HIV-2 strains from INI naïve patients showed the presence of both primary and secondary resistance mutations.

**Conclusion:**

Exposure to antivirals other than INI does not seem to significantly influence the emergence of mutations implicated in INI resistance. HIV-2 strain might have reduced susceptibility to INI.

## Background

Raltegravir (MK-0518; Isentress, Merck) was the first integrase (IN) inhibitor approved for treatment of HIV infection [1], while other compounds such as GS-9137 [2], S-1360 [3], and L-870,810 [4] are at different stages of development. Raltegravir (RAL) has shown potent and durable antiretroviral activity in both treatment naïve [5,6] and highly experienced HIV-1-infected individuals. Due to its novel mechanism of action, RAL was shown to be effective also against HIV-1 strains resistant to reverse transcriptase (RT), protease (PR) and entry inhibitors, both *in vitro *[7] and *in vivo *[8,9]. However, it has been observed that failure of highly active antiretroviral therapy (HAART) including RAL might be related to the emergence of drug-resistant virus variants [10-15], and amino acid changes associated with resistance to integrase inhibitors (INI) have been reported [16-18]. In particular, Y143R/C, N155H and Q148K/R/H have been identified as primary RAL resistance mutations, usually associated with secondary mutations often already present at baseline [10, 12, and 13]. However, the entire panel of mutations associated with RAL resistance has not been fully ascertained. Nor do we fully understand the potential impact of naturally occurring ancillary mutations with respect to: i) promotion of RAL resistance associated mutations, ii) improvement of the activity of mutated IN and iii) HIV-1 replicative capacity. In addition, it is unclear whether drug pressure on the *Pol *gene by RT and PR inhibitors might influence the emergence of primary or ancillary RAL mutations. Finally, it has been observed that about 20% of new HIV infections are now sustained in Italy by a wide variety of subtype non-B HIV-1 strains and a few HIV-2 strains [19,20]. Thus, it is important to define the variability of the IN gene in treatment-naïve and HAART-experienced patients in different HIV-1 subtypes.

The aims of the study were: i) to evaluate IN variability and polymorphism distribution among patients naïve for RAL treatment; ii) to better understand whether previous HAART treatment not including RAL might be associated with the emergence of mutations conferring resistance to INI; iii) to calculate the genetic barrier of primary and secondary mutations associated with INI resistance in different HIV-1 subtypes.

## Materials and methods

### Patients

IN variability was analyzed using stored plasma samples from 95 consecutive patients infected with HIV-1, as well as four HIV-2 positive patients referred to our Institution in the period December 2008 - December 2009. Patients with no available plasma samples or viral load < 1,000 HIV RNA copies/ml plasma were excluded from the analysis. Eligible patients were stratified on the basis of treatment history as follows: i) HAART-naïve patients, ii) RT and PR inhibitor-experienced but RAL-naïve (RTI/PI-experienced).

### Real-time RT-PCR, RT-PCR and sequencing

HIV-1 plasma RNA levels were determined using the Versant HIV-1 RNA 3.0 Assay (Bayer, NY, USA), while HIV-2 plasma RNA levels were determined according to an in house developed real-time RT PCR [21]. For IN gene sequencing (codons 1-277), a region of the HIV-1 Pol gene was amplified in a nested-RT-PCR using primers Int1F, 5'- CAT GGG TAC CAG CAC ACA CAA AGG-3' and Int1R, 5'-CCA TGT TCT AAT CCT CAT CCT GTC -3' for the first PCR round, while primers Int2F 5'-GGA ATT GGA GGA AAT GAA CAA GTA GAT -3' and Int2R 5'-GCC ACA CAA TCA TCA CCT GCC ATC-3' were used in the second PCR round [12]. The first nested-RT-PCR reaction was performed in 50 μl using the SuperScript™ III Platinum^® ^One-Step qRT-PCR System (Invitrogen, Carlsbad, CA, USA) with the following thermal profile: 30 min at 50°C and 10 min at 95°C for 1 cycle, 1 min at 95°C, 1 min at 52°C and 1 min and 10 sec at 72°C for 50 cycles followed by 10 min at 72°C. The nested PCR reaction was performed in 100 μl using TaqGold and the relevant buffer (Applied Biosystem, Foster City, CA, USA) with the following thermal profile: 10 min at 95°C for 1 cycle, 1 min at 95°C, 1 min at 50°C, and 1 min and 10 sec at 72°C for 30 cycles, followed by 10 min at 72°C [12]. RT and PR genes were sequenced in parallel [22]. Sequencing of amplicons was performed using an ABI PRISM 3100 Genetic Analyzer^® ^(Applied Biosystem, Foster City, CA, USA) with the ABI PRISM™ Big Dye Terminator Cycle Sequencing Reaction kit.

### Sequence analysis

IN, RT, and PR sequences were analyzed with the MEGA 4.0 version software [23]. Sequence distances were calculated using the Simmonic sequence editor (version 1.6) program [24], with the Kimura 2-Parameter as a distance estimated method. Divergence was defined as the mean proportion of nucleotide or amino acid differences between all sequence pairs. In each patient, only the predominant virus strain was taken into account to calculate variability. Integrase variability was also calculated in three functional domains: N-terminal domain (NTD), catalytic core domain (CCD), and C-terminal domain (CTD).

### Genotypic resistance and genetic barrier

Resistance to antiretrovirals was estimated on the basis of the Stanford HIV drug resistance database report (http://hivdb.stanford.edu) and the geno2pheno^® ^report (http://integrase.bioinf.mpi-inf.mpg.de/index.php). Twenty-seven IN positions related to 28 mutations in INI resistant HIV-1 strains were categorized as follows: i) primary mutations (E92Q, F121Y, E138A, G140A/S, Y143R/C/H, S147G, Q148H/R/K, S153Y, N155H, R263K) ii) ancillary mutations (H51Y, T66I, L74A/M/I, Q95K, T97A, E138K, Q146P V151I, E157Q,G163R, I203M, S230R) and iii) mutations with an uncertain role (V72I, T125K, A128Y, K160D, V165I, V201I; http://hivdb.stanford.edu).

The genetic barrier to INI-resistance was calculated in 27 IN amino acid positions with a method previously published by van de Vijver et al. [25]. The smallest number of transitions (scored as 1) or transversions (scored as 2.5) were used to calculate the genetic barrier. The genetic barrier was calculated with the sum of scores obtained for each amino acid position.

### Statistical analysis

Statistical analyses were performed using GraphPad Prism (version 4.0) software (San Diego, CA, USA). To compare the nucleotide and amino acid divergence between groups of patients the Mann Whitney U-test was utilized, while the chi-square test was used for comparing the calculated genetic barrier for major and minor substitutions between groups of patients.

## Results

### Study population

Nucleotide (nt) and amino acid (aa) integrase variability was analyzed in 41 HAART-naïve patients, and 54 RTI/PI-experienced patients. Four HIV-2 strains were also analyzed. Among HIV-1 strains from HAART-naïve patients, 16 were subtype B and 25 were non-B strains (A, no.3; C, no.5; G, no.3; F, no.6; CRF02AG, no.6; CRF01AE, no.1; CRF12BF, no.1;). Among HIV-1 strains from RTI/PI-experienced patients, 19 were subtype B and 35 were subtype non-B strains (A, no.1; A/K no. 2; C, no.2 D, no.3; G, no.3; D/F, no.1; F, no.11; CRF02AG, no.9; CRF01AE, no.2; CRF 19CPX, no.1). For comparison, RT and PR gene sequence variability was analyzed in HAART-naïve and RTI/PI-experienced patients (Table 1). HIV-2 strains were from RTI/PI-experienced patients.

**Table 1:** Conserved and variable amino acid distribution in Integrase sequences and amino acid divergence among RT, PR and IN sequences in HIV-1

Category (strain no.)	Mean nucleotide divergence ± SD			Mean amino acid divergence ± SD			IN conserved amino acid (%)	IN variable amino acid (%)		IN conserved amino acid in three functional domains (%)		
					
	RT gene	PR gene	IN gene	RT gene	PR gene	IN gene		Singleton^a^	Parsimony^b^	NTD (1-50)	CCD (51-212)	CTD (213-277)
HAART-naïve (41)	9.2 ± 2.3	10.5 ± 2.9	8.8 ± 2.5	6.3 ± 1.8	11.3 ± 3.5	7.0 ± 1.8	180 (65.9)	36 (13.2)	57 (20.9)	26 (52.0)	114 (70.4)	40 (62.5)
RT/PI-experienced (54)	9.8 ± 2.5	12.2 ± 3.6	8.3 ± 2.5	9.8 ± 2.6	18.8 ± 7.8	6.5 ± 1.9	170 (62.3)	39 (14.3)	64 (23.4)	26 (52.0)	103 (63.6)	41 (64.1)
HAART-naïve *vs *RT/PI-experienced	p < 0.001	p < 0.001	p < 0.001	p < 0.001	p < 0.001	p < 0.001	ns	ns	ns	ns	ns	ns
HIV-2 (4)	ND	ND	13.0 ± 1.1	ND	ND	7.9 ± 1.4	241(88.3)	9(3.3)	23 (8.4)	39 (78.0)	149 (91.9)	53 (82.8)
HIV-2 *vs *HIV-1			p < 0.001			p < 0.001	p < 0.001	p < 0.001	p < 0.001	p < 0.001	p < 0.001	p < 0.001

SD: standard deviation; ND: not done; ns: not significant; NTD: N-terminal domain; CCD: catalytic core domain; CTD: C-terminal domain; RT: reverse trancriptase; PR: protease; IN: integrase.

^a ^A singleton site contains at least two types of amino acid with, at most, one occurring multiple times.

^b ^Parsimony-informative if it contains at least two types of amino acid, and at least two of these occur with a minimum frequency of two.

^c ^Significant p-values are reported.

### HIV IN, RT, and PR variability

Both IN nucleotide and amino acid variability was higher in HAART-naïve patients with respect to RTI/PI-experienced patients (p < 0.001; Table 1). Conversely, RT and PR nucleotide variability was higher in RTI/PI-experienced patients with respect to HAART-naïve patients (p < 0.001).

Gene variability in the HIV-1 B and non-B subtypes was also analyzed. In subtype B strains, IN amino acid variability was statistically higher in HAART-naïve patients with respect to RTI/PI-experienced patients (6.1 ± 1.5 vs 4.1 ± 1.3; p < 0.001). However, both RT (8.3 ± 2.3 vs 4.5 ± 1.2; p < 0.001) and PR (20.3 ± 7.5 vs 8.9 ± 2.8; p < 0.001) amino acid variability was higher in strains from RTI/PI-experienced patients with respect to HAART-naïve patients. In subtype non-B strains, IN amino acid variability was not statistically different in naïve patients with respect to RTI/PRI experienced patients (6.9 ± 1.9 vs 7.1 ± 1.8; p > 0.05), whereas RT (8.0 ± 2.2 vs 7.0 ± 1.9; p < 0.001) and PR (13.3 ± 6.2 vs 10.8 ± 4.1; p < 0.001) amino acid variability was higher in RTI/PI-experienced patients with respect to HAART-naïve patients.

In Table 1, the number of conserved and variable amino acid residues of the IN gene in each group of virus strains is shown. The number of conserved residues in sequences from HAART-naïve patients and RTI/PI-experienced patients was comparable (Table 1). No differences between singleton sites and parsimony informative sites were observed among the variable residues in all patient groups (Table 1).

When the three functional domains of the IN gene were individually analyzed, the amino acid sequences from HAART-naïve patients were slightly more conserved than sequences from RTI/PI-experienced patients. In the analysis of amino acid variability in the three structural domains for all sequence categories, a higher variability in the NTD with respect to the CCD and the CTD was observed (p < 0.001; data not shown).

### Frequency, distribution and genetic barrier of IN resistance mutations

In Table 2, mutations in positions associated with INI susceptibility are shown. The eight primary mutations associated with RAL or elvitegravir (EGV) resistance were not present in any of the strains from INI-naïve patients.

**Table 2:** HIV-1 and HIV-2 amino acid polymorphisms at positions associated with INI (RAL and EGV) resistance

Mutation categories	Known amino acid substitution	Rate of INI resistance mutations in INI-naïve patients (%)	HIV-1 amino acid substitution		HIV-2 clade A ROD subtype consensus	HIV-2 substitution (4 strains)
					
			HAART-naïve (n = 41)	RTI/PI-experienced (n = 54)		
Primary	E92Q				E	
mutations	F121Y				F	
	E138A				S	T_3_
	G140A/S				G	
	Y143R7C/H				Y	
	S147G				S	
	Q148H/R/K				Q	
	S153Y		**A_1_**		A	
	N155H				N	
	R263K				R	
						
Secondary mutations	H51Y				H	
	T66I				T	
	L74A/M/I	7 (7.4)	I_2_/M_1_	I_4_	I	
	Q95K			**R_1_**	R	
	T97A	2 (2.1)	**S_1_**	A_2_	T	
	E138K			**D_1_**	S	T_3_
	Q146P				N	
	V151I				V	
	E157Q	3 (3.2)	Q_2_	Q_1_	H	
	G163R			**E_2_/N_1_/A_1_**	S	G_2_
	I203M	3 (3.2)	M_2_	M_1_	M	
	S230R/M		**N_4_**	**N_2_**	G	
						
Polymorphic and non-polymorphic mutations	V72I	50 (52.6)	I_22_	I_28_/**D_1_**	I	V_2_
	T125K		**A_19_/M_1_/V_1_**	**A_24_/V_4_/P_1_**	E	D_2_
	A128T				M	
	K160D		**Q_1_/R_1_**	**Q_1_**	N	
	V165I	18 (18.9)	I_8_	I_10_	I	
	V201I	68 (71.6)	I_30_	I_38_	I	

RTI: reverse trancriptase inhibitor; PI: protease inhibitor; INI: integrase inhibitor.

New polymorphisms are reported in boldface.

In the 41 HAART-naïve patients, 6 secondary mutations were found: V72I (22/41, 53.7%), L74A/M/I (3/41, 7.3%), E157Q (2/41, 4.9%), V165I (8/41, 19.5%), V201I (30/41, 73.2%) I203M (2/41. 4.9%) (Table 2). Additionally, five substitutions of unknown significance (T97S, T125A/M/V, S153A, K160Q/R, S230N) were found.

In the 54 RTI/PI-experienced patients, 7 secondary mutations were found: V72I (28/54, 51.9%), L74A/M/I (4/54, 7.4%), T97A (2/54, 3.7%), E157Q (1/54, 1.9%), V165I (10/54, 18.5%), V201I (38/54, 70.4%) and I203M (1/54, 1.9%; Table 2). Besides, seven substitutions of unknown significance (V72D, Q95R, T125A/V/P, E138D, K160Q, G163E/N/A and S230N; Table 2) were found. No statistical difference in the prevalence of secondary mutations between sequences from HAART-naïve and RTI/PI-experienced patients was observed (p < 0.05)

Analysis of codon usage distribution between sequences from HAART-naïve and RTI/PI-experienced patients showed that there was no significant difference in the INI resistance mutations with the exception of position 148 (Table 3). At this position, a significant difference in predominant codon usage (CAA vs CAG) between sequences from HAART-naïve and RTI/PI-experienced patients was shown (p = 0.01).

**Table 3:** Codon distribution and calculated genetic barrier at 27 integrase inhibitor susceptible positions in HIV-1 INI-naïve patients

IN codon position	Substitution	Wild type codon	Codon % distribution		Mutational resistance codon	Lower score	IN codon position	Substitution	Wild type codon	Codon % distribution		Mutational resistance codon	Lower scores
											
			HAART-naïve (n = 41)	RTI/PI-experienced (n = 54)						HAART-naïve (n = 41)	RTI/PI-experienced (n = 54)		
51	H51Y	CAT	98	100	TAT	1	143	Y143C	TAC	90	90	TGC	1
		CAC	2	0	TAC	1			TAT	10	10	TGT	1
66	T66I	ACA	98	94	ATA	1		Y143R	TAC	90	90	CGC	2
		ACC	0	6	ATC	1			TAT	10	10	CGT	2
		ACG	2	0	ATA	2	146	Q146P	CAA	98	98	CCG	2.5
72	V72I	GTT	32	31	ATT	1			CAG	2	2	CCA	2.5
		GTC	15	6	ATC	1		Q146K	CAA	98	98	AAA	2.5
		GTA	0	6	ATA	1			CAG	2	2	AAG	2.5
		GTG	0	4	ATA	2	147	S147G	AGT	76	70	GGT	1
	**I72**	ATT	41	45		0			AGC	24	30	GGC	2
		ATC	12	2		0	148	Q148H	CAA	80	96	CAT/C	2.5
		ATA	0	4		0			CAG	20	4	CAT/C	2.5
	**D72**	GAT	0	2	ATT	3.5		Q148K	CAA	80	96	AAA	2.5
74	L74I	CTG	72	78	ATA	3.5			CAG	20	4	AAG	2.5
		CTA	10	15	ATA	2.5		Q148R	CAA	80	96	CGA	1
		TTA	10	2	ATA	2.5			CAG	20	4	CGG	1
		TTG	2	0	ATA	3.5	151	V151I	GTA	71	72	ATA	1
	**I74**	ATT	0	0		0			GTG	29	28	ATA	2
		ATA	2	7		0	153	S153Y	TCT	76	87	TAT	2.5
	**M74**	ATG	2	0	ATA	1			TCC	15	11	TAC	2.5
92	E92Q	GAA	63	61	CAA	2.5			TCA	7	2	TAT/C	5
		GAG	37	39	CAG	2.5		**A153**	GCC	2	0	TAC	5
95	Q95K	CAA	32	28	AAA	2.5	155	N155H	AAT	90	93	CAT	2.5
		CAG	68	70	AAG	2.5			AAC	10	7	CAC	2.5
	**R95**	CGG	0	2	AAG	3.5		N155S	AAT	90	93	AGT	1
97	T97A	ACA	98	96	GCA	1			AAC	10	7	AGC	1
	**A97**	GCA	0	4		0	157	E157Q	GAA	88	91	CAA	2.5
	**S97**	TCA	2	0	GCA	2.5			GAG	7	7	CAG	2.5
121	F121Y	TTC	90	98	TAC	2.5		**Q157**	CAA	5	2		0
		TTT	10	2	TAT	2.5	160	K160D	AAA	85	93	GAT/C	3.5
125	T125K	ACA	27	18	AAG	2.5			AAG	11	5	GAT/C	3.5
		ACT	10	4	AAA/G	5		**R160**	AGA	2	0	GAT	4.5
		ACG	12	24	AAG	2.5		**Q160**	CAA	2	2	GAT/C	5
	**A125**	GCA	44	33	AAA	3.5	163	G163R	GGA	63	57	AGA	1
		GCG	2	6	AAG	3.5			GGG	27	39	AGG	1
		GCT	0	6	AAA/G	6			GGT	0	2	CGC,AGA/G	3.5
	**V125**	GTG	0	7	AAG	3.5		**E163**	GAA	0	4	AGA	2
		GTA	2	0	AAA	3.5		**N163**	AAC	0	2	CGC,AGA/G	3.5
	**M125**	ATG	2	0	AAG	2.5		**A163**	GCG	0	2	CTG,AGG	3.5
	**P125**	CCG	0	2	AAG	5	165	V165I	GTA	56	52	ATA	1
128	A128T	GCA	54	50	ACA	1			GTC	22	18	ATC	1
		GCC	37	46	ACC	1			GTT	2	10	ATT	1
		GCT	9	4	ACT	1			GTG	0	2	ATA	2
138	E138K	GAA	100	98	AAA	1		**I165**	ATA	20	18		0
	**D138**	GAC	0	2	AAA/G	3.5	201	V201I	GTA	24	28	ATA	1
140	G140S	GGA	46	37	AGT/C	3.5			GTG	2	2	ATA	2
		GGC	44	46	AGC	1		**I201**	ATA	74	70		0
		GGT	5	6	AGT	1	203	I203M	ATA	95	98	ATG	1
		GGG	5	9	AGT/C	3.5		**M203**	ATG	5	2		0
	G140A	GGA	46	37	GCA	2.5	230	S230R	AGC	90	96	AAC	1
		GGC	44	46	GCC	2.5		**N230**	AAC	10	4		0
		GGT	5	6	GCT	2.5	263	R263K	AGA	85	96	AAA	1
		GGG	5	6	GCT/G	2.5			AGG	15	4	AAG	1

The amino acids differing from wild-type or expected mutant are in boldface.

### HIV-2 variability and mutation distribution

HIV-2 strains showed higher conservation with respect to HIV-1 subtype B and non-B strains (p < 0.001). In detail, in HIV-2 strains, 241/273 (88.3%) conserved sites and 32/273 (11.7%) variable sites were observed (Table 1). The analysis of four HIV-2 integrase genes showed that the mean nucleotide and amino acid variability was 13.0% ± 1.1 and 7.9% ± 1.4, respectively. Three out of four HIV-2 strains analyzed (all from RTI/PI-experienced patients but INI naïve) showed the presence of an E to T polymorphism, in a position where a primary mutation (E138A normally associated with RAL-resistant HIV-1 strains) was detected. Three secondary mutations I72V, E125D and S163D (Table 2) were also detected.

## Discussion

The introduction of the new INI antiviral-drug class [6,26] is an important step forward in the treatment of HIV-1 infection [8]. Despite the success of HAART in managing HIV-1 infection, the development and worldwide spread of HIV-1 drug resistant strains remain serious issues. In this study, we analyzed the IN gene variability in parallel with RT and PR variability with the aim of evaluating whether HIV genetic background influences the appearance of IN mutations. The distribution of specific amino acids implicated in INI susceptibility was also compared in different HIV-1 subtypes and in patients naïve for treatment or exposed to RTI and PI. The simultaneous evaluation of RT, PR and IN identity showed that the IN gene had lower amino acid variability. This would confirm the high level of integrase sequence conservation reported by Rhee et al. [27]. Moreover, the analysis of three functional integrase domains showed only a small difference in the catalytic core domain.

The evaluation of IN variability in different patient categories showed no differences in subtype non-B and a higher divergence in subtype B strains from HAART-naïve compared with RTI/PI-experienced patients. These results are in contrast with the previously reported greater amino acid IN divergence in RT/PI-experienced patients [28]. On the other hand, we observed a greater RT and PR amino acid divergence in both B and non-B strains from RTI/PI-experienced patients with respect to HAART-naïve patients. These findings are consistent with the hypothesis that multiple rounds of positive selection by subsequent HAART regimens including different RTI and PI, may lead to the emergence of a wider number of RT and PR variants in contrast with little or no change in the IN gene.

In keeping with previous studies [13,29-32] no primary IN mutations associated with INI susceptibility were present in strains from INI-naïve patients. However, in sequences from INI-naïve patients some secondary mutations, which have been shown to contribute to RAL resistance [28-31], were found. The low prevalence and the equal distribution of these polymorphisms among the different groups of patients are in contrast with the reported appearance of secondary mutations in association with prior antiretroviral exposure [28,31]. Thus, our results are in accordance with findings by Garrido et al., [32] and suggest that the emergence of RAL resistance mutations is weakly influenced by prior exposure to antiretrovirals other than INI.

Finally, new polymorphisms (Q95R, and T97S) in positions related to INI resistance were found. Whether and how these mutations might influence viral fitness or replication remains to be clarified.

The genetic barrier was calculated for 27 amino acid positions related to INI susceptibility. The majority of these positions were highly conserved. Our analysis extends the results reported by Maïga et al [33] (including only B and CRFD02_AG subtypes) to a wider collection of HIV-1 subtypes which reflects the evolving epidemiology of this infection in our region [19]. Analysis of codon usage distribution between sequences from HAART-naïve and RTI/PI-experienced patients revealed a single position (148), with a predominant difference in codon usage. These findings suggest a marginal yet valid influence of prior antiretroviral exposure on the genetic barrier in our study population [33]. A larger dataset would allow better definition of the role played by previous treatment with RTI and PI on INI susceptibility. On the other hand, the great majority of patients on HAART show complete suppression of peripheral viral load, and the enrollment of 95 viremic patients required 1 year to be completed.

Due to the small number of HIV-2 sequences, our analysis did not allow us to draw conclusions on HIV-2 variability. However, of particular interest was the detection of mutations in IN positions associated with RAL resistance. This finding confirms and extends a previous observation by Xu et al. [34]. The identification of INI-resistance mutations in INI-naive patients infected with HIV-2 highlights the urgent need for future studies on HIV-2 and may necessitate avoidance of INI in the treatment of these patients.

In conclusion, primary INI resistance-associated mutations were not present in this population of INI naïve HIV-1 infected individuals. Exposure to antivirals other than INI does not seem to significantly influence the emergence of mutations implicated in INI resistance.

## Competing interests

The authors declare that they have no competing interests.

## Authors' contributions

AP has made great contribution to sequences analysis and manuscript preparation. SP and RG have been involved in sample collection and sequencing. GC has been involved in sample collection. FB has contributed in manuscript preparation and fund raising.

All authors read and approved the final manuscript.
